# A simulation study of drone delivery of Automated External Defibrillator (AED) in Out of Hospital Cardiac Arrest (OHCA) in the UK

**DOI:** 10.1371/journal.pone.0259555

**Published:** 2021-11-15

**Authors:** Nigel Rees, Jeremy Howitt, Nigel Breyley, Phil Geoghegan, Carl Powel

**Affiliations:** 1 Welsh Ambulance Services NHS Trust (WAST): Pre Hospital Emergency Research Unit (PERU), Institute of Life Sciences Swansea University Singleton Park, Swansea, Wales, United Kingdom; 2 Cadair Consulting Ltd, Bristol, United Kingdom; 3 Cyclops Air Cyclops Air Ltd, Lincoln, United Kingdom; 4 SwiftFlight Avionics Ltd, Altrincham, United Kingdom; St. Michael’s Hospital, CANADA

## Abstract

**Background:**

Drones are increasingly used in healthcare, and feasibility studies of deployment of Automated External Defibrillators (AED) in Out-of-hospital cardiac arrest (OHCA) have been conducted. Despite the potential contribution of drones to healthcare, regulatory barriers exist, including limits on flights beyond visual line-of-sight (BVLOS). The aim of this project was to deliver an AED BVLOS in Wales.

**Methods:**

We developed of a Concept of Operations (CONOPS) to identify requirements, constraints, organisation and roles and responsibilities associated with deploying a drone to deliver an AED BVLOS. We equipped a Penguin B drone with satellite-enabled technology to enhance situational awareness and safety for the remote pilot. A BVLOS Operating Safety Case and three-week flight test programme was conducted with an AED attached directly to parachute for deployment to simulated OHCA.

**Results:**

We completed six flights totalling 92km, 1:02.5 hours of flight time and four successful parachute payload drops. We conducted a successful end-to-end flight demonstration of an AED delivered via BVLOS by drone to a simulated OHCA and resuscitation by lay responder’s in a remote location; the final delivery of 4.5km was completed in 2:50 minutes.

**Conclusion:**

We have delivered an AED by parachute, from fixed wing drone BVLOS in the UK in simulated OHCA. This project adds to the body of knowledge required for regulatory assurance on drone use BVLOS. Further research is needed before routine use of this technology.

## Introduction

The use of drones in healthcare is increasingly being explored through applications such as delivering medicines and vaccines [1], blood [2] and other medical supplies that are urgently needed in inaccessible areas [3, 4]. There are many potential benefits for the use of drones in healthcare, which includes overcoming supply challenges frequently caused by poor transport networks, extreme weather conditions, natural disasters or traffic congestion in urban areas [5]. An emerging use by ambulance services includes the deployment of Automated External Defibrillators (AED’s) by drone in Out-of-hospital cardiac arrest (OHCA).

OHCA is the third leading cause of death in industrialised nations with an estimated 275,000 cardiac arrests in Europe each year, but despite many advances in this area, survival remains low [6, 7]. In the UK, average overall OHCA survival is around 8.6%, which is significantly lower than other countries such as Holland, Seattle and Norway where more than 20 of people, who sustain OHCA survive [8–10]. Care recommendations are increasingly emphasizing improvements in Basic Life Support (BLS) training, public education and access to AED’s. These are elements of the Chain of Survival adopted by Save a Life Cymru [11], which represents a series of sequential actions to be taken to improve chances of survival from OHCA.

Survival rates of 50–70% from OHCA can be achieved with defibrillation within 3–5 min of OHCA [12], and probability of survival is reduced by 10% for each minute delay, yet less than 2% of victims have an AED applied before arrival of an ambulance [13]. Responsibility and provision of Healthcare and Ambulance services in Wales is devolved from UK Parliament to the Welsh Government who in in turn commission the Welsh Ambulance Services NHS Trust (WAST). WAST currently achieve an emergency response to 73.8% of red calls (the call category for OHCA) within 8 minutes [14], which is comparable to other UK Ambulance Services [15, 16]. However, within the study area, there are geographical variations in achieving this response, with the most rural county of Powys meeting this target 65.9% of the time versus the most urban area of Cardiff & Vale at 81.3% [14]. Wales has a population of approximately 3 Million people a total area of 2,064,100 hectares, and many inaccessible places such as remote beaches and mountainous terrain. This may result in delays of ambulance response to red calls, significantly more than the 8 minutes target which may not be reflected in the reported figures.

A wide range of initiatives have been introduced in order to reduce delays in deployment of AED in OHCA, and drone technology is increasingly seen as a viable option. Claesson et al (2016, 2017) [17, 18] conducted simulation studies for deployment of AED in rural OHCA, where the drone arrived before the ambulance in 93% of cases, with a median reduction in response time of 16:39 minutes. Claesson et al (2016, 2017) [17, 18] concluded that further test flights, technological development, and evaluation of integration with dispatch centres and aviation administrators are needed.

Drones provide particular potential benefits in deployment of AED’s in remote and inaccessible locations, but key to future viability of their use in this context includes the Civil Aviation Authority (CAA) regulations which do not currently permit drone flights Beyond Visual Line-Of-Sight (BVLOS), primarily due to the inability to visually monitor the airspace for other aircraft, and absence of sense and avoid technology [19]. In its Regulatory Sandbox brief [20], and recent formal principles document (CAP1861) [21], the CAA posed several BVLOS questions that must be addressed in order to improve knowledge and enable the development of appropriate regulatory frameworks and approval mechanisms.

The present paper reports on the progress of a UK Space Agency/Welsh Government technology funded project to conduct a proof-of-concept demonstration showing how satellite-enabled drones could be used as part of a broader satellite-enabled network to support remote healthcare services in rural Welsh communities. The project involved a collaboration with Snowdonia Aerospace, SwiftFlight Avionics, the University of Manchester and Welsh Ambulance Service NHS Trust (WAST). Within this paper we focus on the clinical aims of the project to deliver an AED beyond line of visual sight (BVLOS) in Wales. Our objectives were to complete test flights, successfully achieve payload drops of an AED, and conduct a successful end-to-end flights demonstrating proof-of-concept for the delivery of an AED via drone BVLOS to a remote and rural location difficult to reach by ambulance.

## Methods

This study was not deemed to be NHS research within the Health Research Authority (HRA 2019) [22] requirements which regulates research involving humans in the UK. Other similar drone studies internationally likewise have not met the criteria of such research regulation [15, 23, 24]. However, the present study was conducted in accordance with the requirements of the declaration of Helsinki (1963) [25] and WAST Research and Development forum provided institutional approval and maintained oversight throughout.

Working in partnership, Snowdonia Aerospace, WAST and Swift Flight Avionics gathered a team of aerospace engineers, researchers and technical and ambulance service leaders to develop and deliver this project through the following stages:

### Concept of operations

An outline Concept of Operations (CONOPS) for the use of BVLOS drones to support OHCA events was developed and used to inform the CAA of our intentions in order to gain operating permission. A CONOPS is a user-oriented document that describes systems characteristics for a proposed system in terms of the user needs it will fulfil, its relationship to existing systems or procedures, and the ways it will be used. The CONOPS document identified the requirements, constraints, organisation and roles and responsibilities associated with deploying an ambulance service drone to deliver an AED. Based on this information, the key sequence of events, process flows and actors in the emergency response to an OHCA event in a remote and rural location were identified. The demonstration programme focused on the flight test activity, and was therefore to confirm proof-of-concept. Additionally for the demonstration programme, the external pilot was also responsible for manual take-off and landing under visual line-of-sight (VLOS) rules before handing over to the internal pilot to execute the automatic BVLOS mission.

Our project required a drone capable of flight BVLOS, which led to a limited choice of model of drone. The parachute used was designed for use as an emergency chute for smaller drones but came with a datasheet available with approximate drop rates. We tested this from a static tower at first with a simulated defibrillator weight to check drop rate (speed) and therefore to gain an awareness of potential time to drift with wind. Practice drops were then flown overhead at the aerodrome to validate these figures and calculate a model for best drop height. Drop can be automatically cued by GPS co-ordinates (with allowance for wind) or manually released via radio link. Covid-19 restrictions curtailed time available and so we were unable to gather more data points to validate drop calculations.

### Penguin B vehicle systems installation and integration

Following a process of requirements generation, the following components were selected to enhance situational awareness for the remote pilot and for safety functions related to BVLOS operation. Key satellite-enabled equipment included the Automatic Dependent Surveillance-Broadcast (ADS-B) navigation and surveillance units and the Iridium SATCOM. Additional components were also required to store and release the AED payload:

- ADS-B out (uAvionix SkyEcho 2) and ADS-B in (uAvionix Ping RX 1090)
- SATCOM link (Iridium SBD modems)
- Anti-collision lighting (Aveo Engineering high-intensity strobe)
- 4G Video link (Siretta industrial router) and IP Video camera (Datavideo BC50)
- Payload drop enclosure with release mechanism and parachute AED delivery
- Flight termination engine-kill and parachute recovery system

The Penguin was already equipped with a primary UHF command and control (C2) link operating in the 869.525MHz fixed-frequency narrow-band link and an SBG Systems Ellipse-N Inertial Navigation Unit to support primary flight control that features industrial Microelectromechanical systems (MEMS) inertial sensors and a 72-channel global navigation satellite system (GNSS) receiver (L1 GPS + GLONASS or GPS + BEIDOU). Under-wing locations were selected for the payload drop enclosure and parachute recovery system and in order to achieve the overall vehicle weight and balance requirements many of the internal components had to be moved to new locations.

### Automatic External Defibrillator (AED)

Schiller UK Ltd. provided the FRED easyport AED, which is the world’s first pocket defibrillator and is both ultra-light (only 490 grams, including batteries) and ultra-small (133mm height x 126mm width x 35 mm depth), making it ideal for deployment by drone. We also felt the parachute would make it more visible to those on the ground. The parachute was therefore attached directly to the protective carry case without need for any modification.

### Proposed operating area and BVLOS operating safety case

Our original proposal declared the intent to fly from the Snowdonia Aerospace Centre, Llanbedr, directly across Cardigan Bay to drop the AED; a flight distance of 12 nautical miles. This was based on our BVLOS Operating Safety Case (OSC) 15, as submitted to the CAA on 23rd December 2019. Unfortunately, the CAA advised that as a matter of policy, as they would not permit BVLOS outside of segregated airspace as per the Civil Aviation Authority (CAA) regulations above [19] We were therefore requested to update our initial OSC, and without the potential to incorporate an active detect-and-avoid system within the budget and timescales of the project, we agreed as a further mitigation to constrain operations within the segregated airspace of the temporary danger area and to fly mission profiles down the coast a maximum of 5 nautical miles. Our revised OSC was approved by the CAA on 19th March 2020.

### Flight test and demonstration programme

The Penguin drone was deployed to Snowdonia Aerospace Centre on Thursday 5th March 2020 for a scheduled three-week flight test programme.

## Results

We conducted a simulated OHCA event in a remote and rural location and completed a total of six flights (summarised in S1 File.) with a combined total flight time of 1:02.5 hours and flight distance of 92km. The indicated airspeed of the flight was 25m/s throughout, with a maximum return range of around 50miles in the current configuration. However, we have not benchmarked the range performance (this is from supplier specifications). The flights were conducted at a height of 120 metres/400feet throughout, and the parachute/AED drop height was through deployed at release, and therefore also from 120m. The flights were limited somewhat by the crosswind for take-off of 5 knots. Wind is important, but critical factors for drop precision and feasibility also includes the suitability of the drop area and the proximity of high terrain or obstructions (affects run-in and run-out manoeuvres and selected release height); we did not encounter wind restrictions for the parachute drop.

The flight test summary data is included in S1 File which lists flight time details. These flight times were however short and do not tell the whole story as the AED delivery was a single flight due to COVID-19 pandemic constraints on the timescales, and so this information may be of limited value. We attempted and successfully completed four AED payload drops, the first three were within the confines of the airfield and were all system development tests and the final drop (Flight #6) was made onto the beach at Morfa Dyffryn/Benar and was a representative BVLOS drop.

The AED was deployed to a simulated OHCA scenario involving lay responders who used the AED within their resuscitation attempt. It landed within 50 metres of the target scenario and no damage occurred to the drone nor defibrillator during our tests. The AED when dropped using a parachute, and had a descent rate of 4 metres per second. The drop height is therefore be adjusted to take account of operating location and accuracy required. In theory the lower the drop height the more accurate the delivery (i.e. less drift with the wind) but features such as terrain and radio signal propagation are factors.

## Discussion

We have achieved the aim of this project and delivered an AED BVLOS in Wales. Whilst Claesson et al (2017) [18] was the first to deliver an AED by drone for actual BVLOS flights, we believe this is amongst the first simulated UK delivery of a healthcare product by drone BVLOS. We also achieved both of our objectives as flight #6 included a complete end-to-end mission of delivery of an AED via BVLOS drone to a remote and rural location that would be difficult to reach with an ambulance in a timely fashion. The delivery leg down the coast to the point of payload release measured 4.5km and was completed in only 2:50 minutes. The approach profile was deliberately offset ~50m laterally from our observers on the beach (the “casualty” and “first aider”) and the drop was triggered automatically. We therefore achieved our objective of completing test flights and successfully achieving payload drops of an AED.

We believe our study has progressed the body of knowledge and technical understanding of the use of fixed wing drone to parachute deploy AED BVLOS in OHCA. We concur with others by recognising the need for further research, innovation and regulatory progress to determine their operational, economic, clinical and cost effectiveness when integrating into emergency medical and wider health care systems [26, 27]. Studies on use of drones in OHCA continue to report that it is feasible and acceptable in a community setting, providing more timely access to early defibrillation [28]. Studies also continue to explore the retrieval and use of an AED delivered via drone and report positive experiences from participants interacting with a drone during OHCA in a community setting [28, 29]. Mackle et al (2020) [30] recently linked real world datasets in Northern Ireland and built a system to determine the difference in emergency response times when having aerial ambulance drones available compared to response times when depending solely on traditional ambulance services and lay rescuers who would use nearby publicly accessible defibrillators to treat OHCA victims. Mackle et al (2020) [30] found that after the drone network was implemented, publicly accessible AEDs made up 19.74% of responses, ambulances made up 25.66% and drones made up 54.6% of total responses. Such studies along with ours are thus continuing to build the knowledge base for application and adoption of drones in this context.

We recommend that future work should explore parachute dropping an instrumented payload, for several identical flights, to accurately determine the drop trajectory, dispersal statistics and enable optimisation of the drop accuracy. However, as the most significant driver for drop accuracy is the height above the surface at the point of release, this should be minimised, but there are differing limiting characteristics at each drop location due to varying terrain, obstacles and communications coverage.

We will conduct future studies to deploy an AED BVLOS and have since secured funding aiming to accelerate development and testing of an active detect-and-avoid solution for light drones, typically less than 150kg take-off weight, enabling their safe and full integration into the UK aviation system. Our aim is to achieve approval for regular and routine beyond visual line-of-sight (BVLOS) operations in non-segregated airspace by the end of the project. We will also continue to explore with stakeholders opportunities and barriers to the implementation of drones into healthcare.

## Limitations

Unfortunately unseasonably high winds (compared to historical record for the aerodrome) and the emerging COVID-19 pandemic meant that we had to significantly compress the test programme. The test team was however able to observe all Government guidelines on social distancing and complete a successful end-to-end mission that showed proof-of-concept for delivery of an AED via BVLOS drone to a remote and rural location before the programme was prematurely closed on Monday 23rd March to comply with the UK-wide COVID-19 lockdown.

Our study aims and objectives were very specific, focusing on many of the technical and regulatory challenges of deploying an AED BVLOS in Wales. Our original proposal intended to fly across Cardigan Bay to drop the AED; a flight distance of 12 nautical miles. Unfortunately, the CAA advised that as a matter of policy, that they would not permit BVLOS outside of segregated airspace and requested that we update our initial OSC. This limitation highlights how our study is informing the regulatory journey required before the full potential of drones can be achieved in healthcare which will undoubtedly require them to fly BVLOS.

The AED drop had been planned based on the 180/10kt wind that had been recorded at the airfield, but actual conditions at the drop site were light and variable with near zero wind, which resulted in the payload landing ~60m downrange (south) from observers. It is expected that updating wind reference data closer to the drop time and further refinements in the release logic could lead to a ~15m Circular Error Probability (CEP) at the drop location—*i*.*e*. the radius of a circle where the probability of the touchdown point being inside is 50%. There were many implementation issues in terms of wind and rain and many days that flights did not occur. These are significant limitation which are especially important in this area and will need to be addressed in future studies exploring feasibility of using drones in this context.

Our project does not therefore fully address wider questions on how such technology would integrate into the health care systems to provide equitable access to such a service. Our study does not also provide answers on clinical and cost effectiveness of AED’s deployed by drones BVLOS, and we are not aware of any such published studies to have done so?

## Conclusion

We have demonstrated the safe simulated delivery of an AED BVLOS by drone in Wales (UK). Our study has addressed many of the technical, logistical, geographical, and regulatory challenges involved in this process, and provides a significant contribution to the body of knowledge in this area, which will aid in building the assurances required by regulators and others. Our study provides particularly interesting insights on the use of fixed wind drone and parachute AED drop.

Harnessing the potential of drone technology in the care of OHCA may become one of several innovative solutions to improve access to earlier defibrillation and result in the saving of many lives? This simulated use of drones in OHCA may lead to further adoption of their use into the wider healthcare system, bringing potential improvements in the efficiency, effectiveness and quality of care? Despite this, further research, innovation and regulatory approvals will be required before routine adoption of this technology into UK healthcare.

## Supporting information

S1 File (DOCX)
