# Supplementary material for: A simulation study of drone delivery of Automated External Defibrillator (AED) in Out of Hospital Cardiac Arrest (OHCA) in the UK
Source: PLoS One. 2021 Nov 15;16(11):e0259555. doi: 10.1371/journal.pone.0259555 (PMC8592459; doi:10.1371/journal.pone.0259555)
Supplement: S1 File — (DOCX) [file pone.0259555.s001.docx]

**Test Flight Summary:**
